# Efficacy and safety of transcatheter closure in adults with large or small atrial septal defects

**DOI:** 10.1186/s40064-016-3552-z

**Published:** 2016-10-21

**Authors:** Matthias R. Meyer, David J. Kurz, Alain M. Bernheim, Oliver Kretschmar, Franz R. Eberli

**Affiliations:** 1 Division of Cardiology, Department of Internal Medicine, Triemli City Hospital, Birmensdorferstrasse 497, 8063 Zurich, Switzerland; 2 Division of Pediatric Cardiology, Pediatric Heart Centre, University Children’s Hospital, Zurich, Switzerland

**Keywords:** Atrial septal defect, Congenital heart disease, Device size, Percutaneous transcatheter closure

## Abstract

**Background:**

In most patients with secundum atrial septal defects (ASD), transcatheter closure is the preferred treatment strategy, but whether device size affects clinical outcomes is unknown. We sought to study the efficacy and safety of large closure devices compared to the use of smaller devices.

**Methods:**

Using a single-center, prospective registry of adult patients undergoing transcatheter ASD closure, patients receiving a large closure device (waist diameter ≥25 mm, n = 41) were compared to patients receiving smaller devices (waist diameter ≤24 mm, n = 66). We analyzed pre-interventional clinical, hemodynamic and echocardiographic data, interventional success and complication rates, and 6-month clinical and echocardiographic outcomes. The primary efficacy outcome was successful ASD closure achieved by a single procedure and confirmed by lack of a significant residual shunt at 6 months. The primary safety outcome was a composite of device embolization, major bleeding, and new-onset atrial arrhythmia occurring within 6 months.

**Results:**

Transcatheter ASD closure using large devices was successful in 90 % compared to 97 % of patients receiving smaller devices as defined by the primary efficacy outcome (p = 0.20). The primary safety outcome occurred in 4 patients of the large and 6 patients of the small device group, resulting in an event-free rate of 90 and 91 %, respectively (p = 0.89). Similar significant symptomatic improvement was observed in both treatment groups after 6 months, indicated by a 50 % increase in the fraction of patients in NYHA class I (p < 0.0001 vs. baseline).

**Conclusions:**

Transcatheter closure in this cohort of patients with large or small ASD was effective with similar complication rates during short-term follow-up irrespective of the size of the implanted device.

## Background

Atrial septal defects (ASD) are the most common congenital heart defects diagnosed in adults. If left unrepaired, an ASD may lead to right ventricular volume overload, pulmonary hypercirculation, and congestive heart failure (Warnes et al. 2008; Baumgartner et al. 2010). Since the first transcatheter device closure of an ASD was performed in 1976 (King et al. 1976), it has become the preferred treatment strategy for isolated secundum ASD due to its high efficacy and low complication rate compared to surgical repair (Du et al. 2002; Thomson et al. 2002; Fischer et al. 2003; Butera et al. 2006), and is now widely used in both children and adults (Baumgartner et al. 2010; Warnes et al. 2008).

Rarely, transcatheter ASD closure may lead to major complications, including perforation of the atrial wall, device embolization, and atrial arrhythmia (Amin et al. 2004; Butera et al. 2006; Du et al. 2002; Fischer et al. 2003; Majunke et al. 2009; Thomson et al. 2002). Although these adverse events occur in less than 2 % of patients, complication rates may increase if large devices are needed for defect closure (Lopez et al. 2005; Butera et al. 2008). In fact, successful device deployment may become technically challenging with less residual tissue surrounding a large ASD (Butera et al. 2008; Guan et al. 2008), and smaller rims as well as the use of large devices may increase the risk of atrial perforation (Amin et al. 2004). In addition, transient atrioventricular (AV) block has been reported after closure of large ASD (Suda et al. 2004; Guan et al. 2008; Marini et al. 2012).

Despite concerns about the safety of large closure devices (Amin et al. 2004; Guan et al. 2008; Lopez et al. 2005), a systematic comparison to the use of smaller devices has not yet been reported. Furthermore, current guidelines do not discuss the potentially increased risk related to transcatheter closure of large as opposed to smaller ASD (Warnes et al. 2008; Baumgartner et al. 2010). The aim of the present study, therefore, was to assess the efficacy and safety of transcatheter ASD closure in patients requiring a large device compared to patients in whom the use of smaller devices was appropriate.

## Results

### Patient characteristics

A total of 107 patients undergoing transcatheter ASD closure were followed through 6 months and analyzed; of these, 41 (38 %) received a large and 66 (62 %) received a small closure device. Clinical characteristics prior to ASD closure are given in Table 1. Patients were between 19 and 82 years of age (mean 48.9 ± 15.6 years), with 57 % being females. 39 % of patients reported symptoms of heart failure according to New York Heart Association (NYHA) functional class II or greater, with no difference between groups. Rates of atrial fibrillation were similar before implantation of large or small devices (17 and 9 %, respectively, p = 0.24). The presence of hypertension, diabetes, dyslipidemia and history of stroke or transient ischemic attack (TIA) also did not differ significantly between groups.

**Table 1:** Baseline characteristics of patients undergoing transcatheter ASD closure

	All patients (n = 107)	Large device group (n = 41)	Small device group (n = 66)	p value
Demographic characteristics				
Age (years)	48.9 ± 15.6	48.9 ± 14.7	48.9 ± 16.3	0.99
Gender (female), n (%)	61 (57)	22 (54)	39 (59)	0.69
Medical history, n (%)				
NYHA I	65 (61)	26 (63)	39 (59)	0.69
NYHA II	32 (30)	11 (27)	21 (32)	0.67
NYHA III	9 (8)	3 (7)	6 (9)	1.00
NYHA IV	1 (1)	1 (2)	0 (0)	0.38
Atrial fibrillation	13 (12)	7 (17)	6 (9)	0.24
Right axis deviation on ECG	11 (9)	8 (20)	2 (3)	0.007
Arterial hypertension	26 (24)	10 (24)	16 (24)	1.00
Diabetes mellitus	4 (4)	3 (7)	1 (2)	0.16
Dyslipidemia	20 (19)	6 (15)	14 (21)	0.45
Stroke/TIA	23 (21)	5 (12)	18 (27)	0.09
ASD characteristics				
Defect size (mm)	24 (16, 27)	27 (24, 34)	19 (14, 24)	<0.0001
Multiple defects, n (%)	9 (8)	3 (7)	6 (9)	1.00
Device size (mm)	24 (18, 28)	30 (26, 34)	20 (16, 24)	<0.0001

Data are given as mean ± standard deviation or median (inter-quartile range) where appropriate. p values were calculated based on statistical comparison of the large and small device groups

*NYHA* New York Heart Association functional class, *ECG* electrocardiogram, *TIA* transient ischemic attack

### ASD size and hemodynamic severity

Median ASD size was 27 mm in patients receiving large and 19 mm in patients receiving small ASD closure devices, with no difference in the rate of multiple defects (Table 1). Median size of implanted devices was 30 and 20 mm, respectively (Table 1). Prior to ASD closure, complete invasive hemodynamics and oximetry data were obtained in 49 patients (Table 2). Patients with large ASD had greater left–right shunt (59 vs. 35 %, p = 0.0002) and pulmonary to systemic flow (Qp/Qs) ratio (2.13 vs. 1.50, p = 0.03). Hemodynamic consequences of large ASD’s were further evidenced by significantly higher right atrial, right ventricular, systolic and mean pulmonary artery as well as pulmonary capillary wedge pressures compared to patients with small ASD (Table 2).

**Table 2:** Shunt calculations and invasive hemodynamics before ASD closure

	All patients (n = 49)	Large device group (n = 19)	Small device group (n = 30)	p value
Shunt calculations				
Qp:Qs	1.57 (1.37, 2.32)	2.13 (1.49, 2.87)	1.50 (1.22, 2.00)	0.03
Left–right shunt (%)	40 (31, 59)	59 (46, 67)	35 (27, 43)	0.0002
Right–left shunt (%)	4 (0, 8)	6 (1, 10)	4 (0, 7)	0.20
Invasive hemodynamics (mmHg)				
RA mean pressure	6 (4, 9)	7 (5, 11)	5 (3, 8)	0.03
RV end-diastolic pressure	8 (6, 10)	9 (8, 12)	6 (5, 8)	0.002
PA systolic pressure	32 (27, 37)	35 (30, 45)	29 (25, 34)	0.004
PA diastolic pressure	11 (8, 15)	11 (7, 16)	11 (8, 15)	0.56
PA mean pressure	19 (15, 24)	24 (16, 28)	18 (14, 22)	0.03
PCWP	11 (7, 15)	14 (11, 16)	9 (7, 13)	0.04
Transpulmonary gradient	9 (5, 11)	10 (8, 11)	9 (4, 11)	0.27

Shunt calculations were performed based on oximetric data using the Fick formula (Miller et al. 1974). Data are given as median (inter-quartile range)

*Qp:Qs* pulmonary to systemic flow ratio, *RA* right atrium, *RV* right ventricle, *PA* pulmonary artery, *PCWP* pulmonary capillary wedge pressure

### Efficacy of transcatheter ASD closure

Successful transcatheter ASD closure was achieved with a single procedure and confirmed by lack of a significant shunt detectable by transesophageal echocardiography (TEE) at 6-month follow-up in 90 and 97 % of patients receiving large and small devices, respectively, with no statistical difference between groups (p = 0.20, Table 3). When only patients receiving Amplatzer devices were included in the analysis, 92 and 97 % of large and small ASD were successfully closed, respectively (p = 0.37). Significant shunts were present following implantation of 3 large and 1 small closure devices (p = 0.16); two patients with large ASD successfully underwent a second transcatheter procedure, while the remaining two patients are being followed clinically due to lack of hemodynamic significance based on repeat invasive assessment or due to concomitant medical conditions. Complete endothelialization of ASD closure devices as confirmed by lack of any residual shunt detected during follow-up TEE was not significantly more frequent in patients receiving small devices (82 vs. 66 % in the large device group, p = 0.07). One patient in the large device group was referred for surgical ASD closure due to device embolization, while a repeat transcatheter ASD closure was performed in one patient in the small device group after distal embolization of the initially implanted device. One patient in each group received 2 devices during the first intervention in order to achieve complete ASD closure.

**Table 3:** Efficacy outcomes 6 months after transcatheter ASD closure

	All patients (n = 107)	Large device group (n = 41)	Small device group (n = 66)	p value
Primary efficacy outcome, n (%)	99 (93)	37 (90)	64 (97)	0.20
Secondary efficacy outcomes, n (%)				
Repeat transcatheter closure	3 (3)	2 (5)	1 (2)	0.56
Referral for surgical closure	1 (1)	1 (2)	0 (0)	0.38
Any residual shunt	26 (24)	14 (34)	12 (18)	0.07

The primary outcome was defined as successful transcatheter ASD closure achieved with a single procedure and confirmed by lack of a significant shunt detectable by TEE at 6-month follow-up as specified in the "Methods" section

**Fig. 1:**
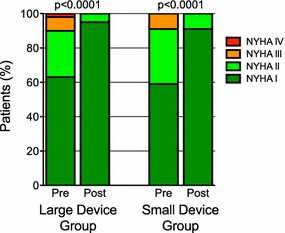
Symptomatic status according to NYHA classification at baseline (*Pre*) and 6 months after (*Post*) transcatheter ASD closure with large or small devices. *NYHA* New York Heart Association functional class

Six months after ASD closure, similar symptomatic improvement was observed in both treatment groups (Fig. 1). Compared to baseline functional status, the fraction of patients in NYHA class I increased from 63 to 95 % and from 59 to 91 % after implantation of large and small devices, respectively (50 % increase, p < 0.0001 vs. baseline). The remaining 5 and 9 % of patients, respectively, were in NYHA class II after ASD closure. Functional status both before the intervention and 6 months thereafter was not different between groups.

### Safety of transcatheter ASD closure

**Fig. 2:**
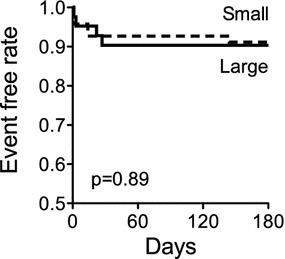
Event-free rate through 6 months after transcatheter ASD closure. Shown are Kaplan–Meier estimates for patients who received a large or small ASD closure device. Adverse events included device embolization, new-onset atrial arrhythmia, and major bleeding graded as Type 2 or greater according to the BARC definition

The primary safety outcome at 6-month follow-up, a composite of device embolization, new-onset atrial arrhythmia, and major bleeding, did not differ between patients receiving large or small devices (10 vs. 9 %, p = 1.0, Table 4). Similarly, there was no difference in the corresponding event-free rates based on Kaplan–Meier estimates between groups (90 vs. 91 %, p = 0.89, Fig. 2). In addition, the primary safety outcome was similar when only patients receiving Amplatzer devices were included in the analysis (11 vs. 10 %, respectively, p = 1.0). In 1 patient, closure of a large ASD (defect size 34 mm) requiring implantation of 2 devices resulted in embolization of the second device into the pulmonary artery. This patient underwent urgent surgical device removal and ASD patch closure. Furthermore, 1 patient presented with acute limb ischemia due to device embolization into the aortic bifurcation 143 days after implantation of a small closure device; this patient underwent surgical device removal followed by a successful repeat transcatheter ASD closure.

**Table 4:** Safety outcomes 6 months after transcatheter ASD closure

	All patients (n = 107)	Large device group (n = 41)	Small device group (n = 66)	p value
Primary safety outcome, n (%)	10 (9)	4 (10)	6 (9)	1.00
Secondary safety outcomes, n (%)				
Device embolization	2 (2)	1 (2)	1 (2)	1.00
New-onset atrial arrhythmia	5 (5)	3 (7)	2 (3)	0.37
Major bleeding	3 (3)	0 (0)	3 (5)	0.28
Death	1 (1)	1 (2)	0 (0)	0.38

The primary safety outcome at 6-month follow-up was a composite of device embolization, new-onset atrial arrhythmia, and major bleeding classified as Type 2 or greater according to the BARC definition

Intervention-related major bleeding complications (bleeding Type 3a according to the Bleeding Academic Research Consortium (BARC) classification (Mehran et al. 2011) at the access site in two patients, and transient hemoptysis in one patient due to guidewire (diameter, 0.018 in.)-related perforation of the left upper pulmonary vein used with a Solysafe device) were observed after implantation of 3 small closure devices, whereas no bleeding complications occurred in the large device group (p = 0.28, Table 4). There was no difference between the large device and the small device groups with regard to fluoroscopy time (15 (11, 20) vs. 14 (11, 23) min), amount of contrast medium used (185 (144, 235) vs. 150 (110, 215) mL), and length of hospital stay (2 (2, 2) vs. 2 (2, 2) days, all data expressed as median and inter-quartile range).

During follow-up, 5 % of patients developed new-onset atrial flutter or fibrillation following ASD closure, with no difference between groups (p = 0.37, Table 4). One patient with a large ASD died during follow-up of a cause unrelated to the closure procedure.

## Discussion

Transcatheter closure is considered first-line therapy over surgical closure for patients with morphologically suitable secundum ASD (Warnes et al. 2008; Baumgartner et al. 2010). In particular, defect closure should be attempted if there is evidence for right ventricular volume overload (Warnes et al. 2008; Baumgartner et al. 2010). However, current guidelines do not take into account whether transcatheter closure of large, hemodynamically more significant ASD may pose an increased risk compared to device closure of smaller defects (Warnes et al. 2008; Baumgartner et al. 2010). Here, based on the first systematic analysis of a cohort of adult patients, we report that implantation of large ASD closure devices is effective with similar complication rates at implantation and through short-term follow-up at 6 months when compared with the use of small devices.

The Amplatzer family of closure devices is widely used for transcatheter ASD therapy. In a pivotal multicenter trial including 596 patients, the Amplatzer Septal Occluder revealed equivalent success rates compared to surgical repair (Du et al. 2002). However, complication rates were lower and the length of hospital stay was shorter in the transcatheter closure group (Du et al. 2002). Subsequent studies have confirmed the safety and efficacy of the device (Thomson et al. 2002; Fischer et al. 2003; Majunke et al. 2009). In the present study, the great majority of patients (93 %) received Amplatzer devices, and we experienced similar high success and low adverse event rates, including device embolism, new-onset atrial arrhythmia, and major bleeding complications as reported previously (Du et al. 2002; Thomson et al. 2002; Fischer et al. 2003; Spies et al. 2008; Humenberger et al. 2011; Majunke et al. 2009).

Currently, devices for closure of defects up to 40 mm are available, although no systematic comparison between the use of large and small devices has yet been performed. Low complication rates following closure of large ASD (median device to septal length ratio: 0.95) in 51 children have been reported (Marini et al. 2012), and there are small series of successful use of Amplatzer Septal Occluders with a waist diameter ≥30 mm in adults (Lopez et al. 2005; Guan et al. 2008). However, concerns about the safety of large devices remain (Lopez et al. 2005). In particular, the most feared complications, such as device embolization and atrial perforation, might be associated with device size (Amin et al. 2004; Guan et al. 2008; Lopez et al. 2005). Smaller rims surrounding a large defect may technically complicate successful device placement thus increasing the risk of embolization (Guan et al. 2008). In our series, two cases of device embolization occurred, one each in the large and small device group. Furthermore, deficient anterior–superior rims of large ASD may increase the chance of contact between the device and the atrial wall, particularly in case of device oversizing, which may result in atrial perforation (Amin et al. 2004). Most atrial perforations occur within the first 3 months after device implantation (Amin et al. 2004), and have also been reported after implantation of small closure devices (Amin et al. 2004; Taggart et al. 2011). In our study, we recorded no evidence of atrial erosion at 6 month follow-up, but obviously, we cannot address the long-term safety of large compared to small devices. Since late atrial perforations have been described (Taggart et al. 2011; Herren et al. 2015), longer follow-up studies would be desirable.

Major bleeding complications related to the closure procedure did not differ between groups. In line with a previous study (Spies et al. 2008), the rate of new-onset atrial arrhythmia during follow-up was also unrelated to device size. In contrast to others (Suda et al. 2004; Guan et al. 2008; Marini et al. 2012), we observed no AV block following the procedure. Overall, this resulted in similar event-free rates in the large and small device group.

In addition to the similar safety outcomes between groups, we observed no difference with regard to successful transcatheter ASD closure achieved by a single procedure and confirmed by lack of a significant shunt detectable by TEE at 6-month follow-up. Initial trivial shunting across the membrane of the occluding device is common and typically closes after full endothelialization of the surface, which was complete within weeks in experimental studies (Lock et al. 1989) but may take longer in some patients (Greutmann et al. 2009; Chen et al. 2011). Although the degree of endothelialization may depend on device diameter (Greutmann et al. 2009), we observed no significant difference in residual shunting following implantation of large or small closure devices. Significant shunting following device implantation was rare, but slightly more common in patients with large ASD, although this did not reach statistical significance. However, large residual shunts in two patients with a large closure device were successfully treated with a second procedure.

In our study, about 40 % of patients reported symptoms of heart failure pre-interventionally. Interestingly, the presence of symptoms was not related to defect size, despite the greater hemodynamic severity shown in the large closure device group. Both treatment groups experienced similar and highly effective symptom improvement after the intervention. However, symptoms commonly aggravate with age, even in the presence of smaller defects (Humenberger et al. 2011). This may be the result of age-dependent diastolic dysfunction (Redfield et al. 2003) or ASD-related impairment of left ventricular compliance (Booth et al. 1988) and thus may not necessarily be related to the size of the ASD.

The main limitations of this study are inherent to its registry design and the relatively small patient number with limited power to detect subtle differences in the rate of rare, but possibly serious adverse events. Given that the efficacy outcomes and some of the safety outcomes show a trend in favor of the small device group, a larger population or meta analysis would be required to demonstrate potentially significant, albeit small effects. An additional bias may represent that 5 patients (4 %) in our study were lost to follow-up. Moreover, Solysafe septal occluders were implanted in 11 patients before an urgent safety notice was issued by the manufacturer (Kretschmar et al. 2010; Knirsch et al. 2011). These patients are being followed by fluoroscopy (Knirsch et al. 2011). In line with the incidence reported by others (Gielen et al. 2012), one wire fracture was diagnosed 2½ years after implantation. No clinical complication has yet resulted in that patient.

## Conclusions

The present study is the first systematic analysis on the use of large compared to small devices for transcatheter ASD closure, a procedure widely performed in adults. In our experience, transcatheter ASD closure using large or small devices is successful and effective with similar complication rates during implantation and short-term follow-up. Provided that sufficient residual septal tissue remains for safe anchorage of the closure device, large ASD can safely be closed by the transcatheter approach.

## Methods

### Patients

Between February 2002 and October 2013, 112 adult patients undergoing transcatheter closure of secundum ASD at the Triemli City Hospital, Zurich, Switzerland, were included in a prospective registry. Indications for closure included dilation of right heart chambers, a left–right shunt with a Qp/Qs ratio of ≥1.5:1, history of paradoxical embolism, and the presence of symptoms such as dyspnea or palpitations in patients with smaller shunts. Detailed medical history, physical examination, and an electrocardiogram were obtained, and the morphology of the ASD was assessed by transthoracic echocardiography and TEE prior to the intervention. If the size and morphology of the secundum ASD precluded the use of a percutaneous device, surgical repair was recommended and patients were not included in the registry. Specifically, if stretched defect size was ≥36 mm, if there was lack of adequate atrial septal rims to permit stable device deployment, especially towards the aortic root, or if the defect was too close to the AV valves, the coronary sinus, or the vena cava (Webb and Gatzoulis 2006), percutaneous closure was not attempted. The study was carried out in compliance with the Helsinki Declaration and approved by the local ethics committee (Kantonale Ethikkommission Zürich). All patients gave written informed consent.

### Hemodynamics and shunt calculations

In about half of the patients, right heart catheterization was performed to obtain complete hemodynamic assessment and oxygen saturations in the right heart chambers and the pulmonary artery, with simultaneous left heart catheterization being performed for shunt quantification by oximetry according to the Fick formula (Miller et al. 1974). In patients >65 years of age, transient ASD occlusion with simultaneous measurement of left sided filling pressures was performed to exclude hemodynamically relevant left ventricular diastolic dysfunction or restrictive disease (Gruner et al. 2012).

### Selection of large or small closure devices

In order to choose the appropriate device size, ASD diameter was determined in all patients before defect closure using intracardiac echocardiography (19 % of patients), fluoroscopy after occlusion of the defect with a sizing balloon (4 % of patients), or both sizing techniques (77 % of patients). ASD closure devices were selected on the basis of these measurements and ASD morphology. For analysis, patients were assigned to two groups receiving either a large or small device. The large device group included patients receiving the following closure devices: Amplatzer Septal Occluder, waist diameter ≥26 mm (n = 32, St. Jude Medical, St. Paul, MN, USA); Amplatzer Multi-Fenestrated Septal Occluder Cribriform, disc diameter 35 mm (n = 6, St. Jude Medical); and Solysafe Septal Occluder, waist diameter ≥25 mm (n = 4, Swissimplant, Solothurn, Switzerland). The small device group included patients receiving the following implants: Amplatzer Septal Occluder, waist diameter ≤24 mm (n = 54, St. Jude Medical); Amplatzer Multi-Fenestrated Septal Occluder Cribriform, disc diameter 25 mm (n = 3, St. Jude Medical); Amplatzer PFO Occluder, right atrial disc diameter 25 mm (n = 5, St. Jude Medical); Solysafe Septal Occluder, waist diameter ≤20 mm (n = 7, Swissimplant); and Helex Septal Occluder, device diameter 25 mm (n = 1, Gore Medical, Flagstaff, AZ, USA). The criteria for device selection at our institution changed over time based on availability and current knowledge about efficacy and safety profiles.

### Transcatheter ASD closure

Device implantation was performed under fluoroscopic guidance and routine intracardiac echocardiography monitoring as described (Greutmann et al. 2009; Gruner et al. 2012). During the procedure, standard heparin (70 IU/kg) was administered intravenously. Cefuroxim (1.5 g intravenously) was used for antibiotic prophylaxis prior to as well as 8 and 16 h after the intervention. Antithrombotic regimen consisted of clopidogrel (600 mg loading dose followed by 75 mg daily) for 3 months along with acetylsalicylic acid (100 mg daily) for 6 months. Endocarditis prophylaxis was recommended for 6 months after ASD closure.

### Follow-up

Six months after ASD closure patient history was obtained, and a clinical examination, electrocardiography, and assessment of the closure device by TEE were performed. For detection of residual shunts Color Doppler imaging and intravenous administration of agitated saline was used during TEE. The presence of a significant residual shunt was diagnosed if >20 microbubbles crossed from the right to the left atrium within the first three cardiac cycles from right-sided contrast opacification while patients were performing a Valsalva maneuver. Follow-up was completed in 107 out of 112 patients (96 %), which were included in subsequent analyses. Complete follow-up was unavailable in 1 patient who received a large closure device (Amplatzer Septal Occluder), and in 4 patients with small closure devices (3 Amplatzer Septal Occluders and 1 Amplatzer PFO Occluder), because they withdrew consent for either undergoing TEE or any follow-up examination.

### Efficacy and safety outcomes

The primary efficacy outcome was defined as successful transcatheter ASD closure achieved by a single intervention, confirmed by lack of a significant shunt detectable by TEE at 6-month follow-up. Secondary efficacy outcomes included need of a repeat transcatheter ASD closure intervention, referral for surgical ASD closure due to failure of transcatheter closure, and presence of any residual shunt detected by TEE. In addition, functional status according to the NYHA classification was assessed at baseline and 6 months after the closure procedure. The primary safety outcome at 6-month follow-up was a composite of device embolization, new-onset atrial arrhythmia, and major bleeding defined as Type 2 or greater according to the BARC definition (Mehran et al. 2011). Secondary safety outcomes included the individual components of the primary safety outcome, as well as all-cause mortality.

### Statistical analyses

The distribution of continuous variables was assessed by D’Agostino and Pearson omnibus normality test. Data with significant deviation from normal distribution were analyzed by the nonparametric Mann–Whitney test and are expressed as median and inter-quartile range. Normally distributed data were analyzed by the unpaired Student’s *t* test and are given as mean ± standard deviation. Categorical data were analyzed by Fisher’s exact test or Chi square test, as appropriate. Kaplan–Meier estimates of event rates were compared between intervention groups with the use of the log-rank test. All analyses were performed using Prism version 5.0 for Macintosh (GraphPad Software, San Diego, CA, USA). p values <0.05 were considered significant.

## Abbreviations

ASD: atrial septal defect
AV: atrioventricular
BARC: Bleeding Academic Research Consortium
ECG: electrocardiogram
NYHA: New York Heart Association functional class
PA: pulmonary artery
PCWP: pulmonary capillary wedge pressure
Qp:Qs: pulmonary to systemic flow ratio
RA: right atrium
RV: right ventricle
TEE: transesophageal echocardiography
TIA: transient ischemic attack
